# Electric-Field-Induced Modulation of Structure and Rheology in MBBA-Based Liquid Crystal Physical Gels

**DOI:** 10.3390/gels12060485

**Published:** 2026-06-01

**Authors:** André Cruz, Andreja Lesac, Nataša Šijaković Vujičić, Francisco J. Galindo-Rosales

**Affiliations:** 1 Transport Phenomena Research Center (CEFT), Associate Laboratory in Chemical Engineering (ALiCE), Department of Chemical and Biological Engineering, Faculty of Engineering, University of Porto, Rua Dr. Roberto Frias, 4200-465 Porto, Portugal; 2 Division of Organic Chemistry and Biochemistry, Ruđer Bošković Institute, Bijenička c. 54, 10000 Zagreb, Croatia; alesac@irb.hr

**Keywords:** liquid crystal physical gel, MBBA, oxalamide gelator, rheology, electrorheology

## Abstract

Liquid crystal physical gels (LCPGs) combine the anisotropic properties of liquid crystals with the structural stability of soft solids. In this work, MBBA-based LCPGs were prepared using chiral oxalamide gelators 1,6-bis((O-leucylmethanol)-N-yloxalamido)hexane (6-O-Me) and 1,9-bis((O-leucylmethanol)-N-yloxalamido)nonane (9-O-Me) and thoroughly characterized for their thermal, rheological, and electrorheological behaviours. Techniques included differential scanning calorimetry, oscillatory rheology, electrorheological testing, and advanced microscopy analysis. A custom microfluidic device was developed for in situ application of an electric field and optical assessment of its influence on microstructure formation. Both gels exhibited distinct gel-like behavior, with storage moduli consistently exceeding loss moduli and sustained network stability under both short- and long-term deformations. The gelators had minimal effect on the isotropic–nematic transition of MBBA but efficiently delayed crystallization, extending the stability window by −8 °C for 9-O-Me and −14 °C for 6-O-Me. When subjected to electric fields, the gel network weakened in the nematic phase, and the fiber assembly during cooling was altered, resulting in the formation of thicker, anisotropic fibers, consistent with microscopic observations. These results illustrate how the properties of LCPGs can be tuned through molecular design and external stimuli, expanding their potential for stimuli-responsive soft matter applications.

## 1. Introduction

Liquid-crystalline physical gels have primarily been developed as electro-optically responsive soft materials for light-scattering displays [1], smart windows [2], and other switchable optical devices [3], where reversible field-induced modulation of transparency is required. Their combination of liquid-crystal anisotropy with mechanically stabilizing supramolecular gel networks additionally makes them attractive for flexible and self-supporting electro-optical materials [4].

Liquid crystals (LCs) are an intermediate mesophase of matter, combining the fluidity of conventional liquids with the orientational order and periodical molecular arrangement of crystals that typifies crystalline solids [5]. This unique state yields materials with anisotropic optical, electrical, and magnetic properties [6].

A gel is defined by two phenomenological characteristics: it should consist of two or more components, one of which is a liquid present in substantial quantity, and it ought to be a soft-solid or solid-like material [7]. In the case of liquid crystalline physical gels (LCPGs), the liquid present in significant quantity is a thermotropic liquid crystal coupled with low percentages of low molecular weight gelators that form self-assembled three-dimensional networks. The self-assembly of the fibrous solid networks of gelators in liquid crystals leads to the formation of soft-solids, keeping the characteristic stimuli-responsiveness of liquid crystals and coupling them with the stabilization of the liquid crystal mesophase by the physical network [8,9]. Typically, the gelators are dissolved in the liquid crystalline mesophase at higher temperatures and form the fibrous network upon cooling [10].

The characteristic thermoreversibility of liquid crystal physical gels leads to the presence of two independent transition temperatures: the isotropic-liquid crystal transition and the sol–gel transition. These transition temperatures are tunable according to the liquid crystal and gelator combination. Furthermore, the sol–gel transition temperature is dependent on gelator concentration, while the isotropic-liquid crystal transition temperature is not affected by that parameter [9].

Liquid crystal physical gels are generally classified into two types depending on the relationship between the sol–gel transition temperature (T_sol–gel_) and the isotropic–liquid crystal transition temperature (T_iso–LC_). In type I gels, gelation occurs in the isotropic phase of the liquid crystal (T_sol–gel_ > T_iso–LC_), leading to the formation of a randomly distributed, isotropic fibrous network that subsequently coexists with the liquid crystalline phase upon cooling. In contrast, type II gels are formed when gelation takes place within the anisotropic liquid crystalline phase (T_sol–gel_ < T_iso–LC_), where the pre-existing liquid crystal order acts as a template, directing the anisotropic self-assembly of the gelator molecules, as shown for the 9-O-Me gelator in HCCA liquid crystal [11].

LCPGs have gained importance for combining the functional anisotropy of liquid crystals with the mechanical stability of soft solids, overcoming the limitations of fluidity and poor mechanical strength in conventional LC materials. Moreover, the solid-like matrix introduced by gelation can enhance alignment stability and reduce flow under external fields, enabling improved performance and broader design flexibility for responsive systems [12].

The physical gelation of room-temperature liquid crystals through the self-assembly of low molecular weight organic molecules via hydrogen bonding was first demonstrated using a trans-1,2-bis(amino)cyclohexane derivative, successfully achieving the gelation of both 5CB and MBBA [13]. Subsequent research has explored a wide variety of gelators and LC hosts, with focus on electro-optical performance, particularly their response time and the relationship between transmittance and applied voltage [9,13,14]. Additionally, advancements in liquid crystal physical gels have been reported, involving the gelation of negative dielectric anisotropy liquid crystals to produce homeotropically oriented nematic physical gels [15]. In comparison with more commonly used cyanobiphenyl-based hosts such as 5CB and E7 [1], MBBA provides a mechanistically attractive model system owing to its negative dielectric anisotropy and well-characterized electro-optical behavior, despite its comparatively lower chemical stability associated with the Schiff-base functionality.

Beyond gelation and electro-optical analysis, further research has emphasized the rheological characterization of these gels. Such studies have demonstrated that these systems, comprising different gelator and liquid crystal pairings, can exhibit high elastic moduli [1,12]. Ruan et al. demonstrated that chirality transfer from molecular gelators to helical supramolecular fibrillar networks can significantly enhance the physical gel performance of liquid-crystalline, enabling unusually low critical gelation concentrations together with high modulus and low driving voltage [16].

There is a growing scientific and technological interest in the design of materials whose internal architecture and, consequently, anisotropic properties, can be actively controlled by external fields during synthesis or operation. In the case of liquid crystal physical gels, the application of electric fields not only enables fine-tuning of their viscoelastic response but also promotes the formation of directionally organized supramolecular structures. Such field-induced anisotropy is essential for the development of next-generation adaptive materials with optimized performance in optics, actuation, and sensing, where precise coupling between molecular alignment and mechanical response is required for reliable device performance [17]. While electro-optical characterization provides information on field-induced reorientation of the liquid-crystalline phase and conventional rheology describes the viscoelastic properties of the gel network under field-free conditions, neither technique alone may fully resolve how electric-field-driven mesogen reorganization influences the supramolecular gel architecture. Electrorheological characterization provides a direct means of probing the interplay between liquid-crystal ordering and network mechanics under applied electric fields, thereby offering mechanistic insight into whether the gel network behaves primarily as a passive scaffold or exhibits structural responsiveness to liquid-crystal reorientation [9,10,18,19,20].

Despite significant advances in the synthesis, structural characterization, electro-optical performance and rheological analysis of LCPGs, their electrorheological behavior remains largely unexplored. Understanding the electrorheological properties of LCPGs is especially relevant for the development of advanced stimuli-responsive materials. Such knowledge could provide key insights for emergent applications in adaptive optics, soft actuators, damping systems, and tunable devices, where fine control of mechanical response via electric fields is highly desirable.

To address this gap, the present work investigates the electrorheological response of MBBA-based liquid crystal physical gels, elucidating the combined effects of gelation and electric-field stimuli on their viscoelastic behavior.

## 2. Results and Discussion

### 2.1. Gelation Properties

Low-molecular-weight organogelators (LMWOGs) based on oxalamide motifs represent an important class of supramolecular systems capable of forming physical gels through directional noncovalent interactions, predominantly intermolecular hydrogen bonding. These interactions drive the self-assembly of gelator molecules into ordered aggregates, which further organize into fibrous networks responsible for gel formation.

**Scheme 1:**
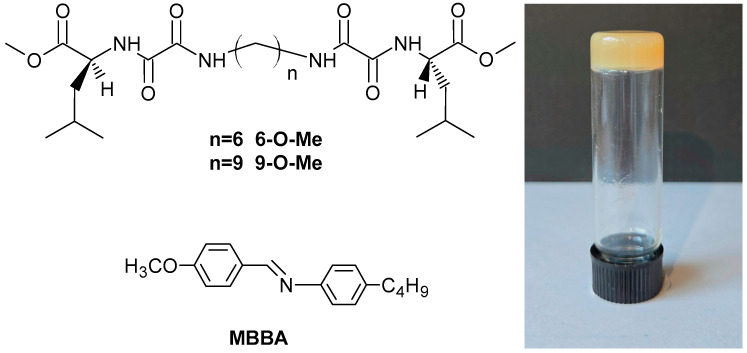
Structures of 6-O-Me, 9-O-Me gelators, MBBA liquid crystal and a photo of 9-O-Me/MBBA gel.

The gelation behavior of the chiral oxalamide gelators 6-O-Me and 9-O-Me in MBBA LC was investigated over the concentration range of 0.3–2.2 wt.%. Both compounds efficiently formed stable liquid-crystalline physical gels throughout the investigated concentration range, demonstrating pronounced gelation ability in the MBBA host (Scheme 1). In all investigated samples, gelation occurred in the isotropic phase of MBBA prior to the isotropic–nematic transition, confirming formation of Type I liquid-crystalline physical gels across the entire studied concentration range.

The investigated gelators contain two oxalamide groups that promote directional hydrogen bonding and formation of supramolecular aggregates with relatively high melting enthalpies. At the selected concentration of 1.6 wt.%, DSC analysis revealed gel-related thermal transitions at 67 °C and 51 °C for the 6-O-Me and 9-O-Me systems, respectively, i.e., above the isotropic–nematic transition temperature of MBBA.

Because gelation occurs in the isotropic phase, MBBA initially acts as an isotropic solvent-like medium during gelator self-assembly (Scheme 1). In a previous study [21], chiral bisoxalamide gelators 6-O-Me and 9-O-Me, incorporating chiral amino acid-derived units and flexible methylene spacers, were shown to efficiently transfer molecular chirality into supramolecular organization in different organic solvents and water. As a result, these systems form diverse chiral morphologies, including twisted ribbons and helical fibers, with the spacer length and conformational flexibility playing a key role in directing the packing and overall architecture of the aggregates. TEM analysis showed that gelators 6-O-Me and 9-O-Me form diverse supramolecular morphologies, strongly dependent on solvent polarity. Increasing polarity promotes the formation of wider and multilayered aggregates.

The gelators 6-O-Me and 9-O-Me have previously been reported to exhibit moderate gelation efficiency in a range of organic solvents, including toluene, xylene, tetralin, 2-octanol, and DMSO/water mixtures, where gelation typically occurs at concentrations of approximately 0.5–3 wt.%. In contrast, stable gels in MBBA are obtained at concentrations as low as 0.3 wt.%, indicating particularly efficient self-assembly in the liquid-crystalline medium [21].

The concentration of 1.6 wt.% was selected as an optimized working concentration within the literature-reported range for liquid-crystalline physical gels (typically 0.5–5 wt.%), ensuring isotropic-phase gelation together with formation of a dense fiber network. This concentration additionally provided (i) fibrillar structures clearly observable by optical microscopy and (ii) viscoelastic network strength for reliable electrorheological measurements and clear detection of electric-field-induced rheological changes.

FTIR spectroscopy provided additional evidence for the intermolecular hydrogen-bonding interactions responsible for gel network formation in both 6-O-Me and 9-O-Me liquid-crystalline gels (Supplementary Information Figure S8). As previously demonstrated for the oxalamide gelators in organogel (toluene gel), the positions of NH, amide I, and amide II bands are characteristic of hydrogen-bonded supramolecular assemblies, while the simultaneous appearance of lower-wavenumber hydrogen-bonded and higher-wavenumber free functionalities indicates coexistence of network-assembled and non-associated molecules [21,22]. In our previous organogel studies, xerogel spectra showed broadened NH, amide I, and amide II absorptions at positions corresponding to strongly hydrogen-bonded molecules, confirming preservation of the intermolecular hydrogen-bonding motif in the self-assembled network [21].

A similar spectral pattern was observed in the MBBA liquid-crystalline gels studied here. The 6-O-Me/MBBA and 9-O-Me/MBBA gels exhibited characteristic absorptions at approximately 3290 cm^−1^ (NH stretching), 1740 cm^−1^ (ester carbonyl), and 1655 cm^−1^ (amide I), consistent with hydrogen-bonded oxalamide assemblies forming the gel network. In contrast, the sample prepared under continuous electric-field-assisted cooling from the isotropic phase exhibited additional bands at 3473 cm^−1^ and 1688 cm^−1^, assigned to free NH and free amide functionalities, respectively, while the hydrogen-bonded bands at 3290 and 1655 cm^−1^ remained present. The simultaneous presence of hydrogen-bonded (lower wavenumber) and free (higher wavenumber) functionalities suggests coexistence of strongly network-associated gelator molecules and less associated species generated under electric-field-assisted assembly, in analogy with the behavior previously observed in organogel systems [21].

Importantly, the amide bands in the field-assisted cooling sample were significantly sharper and more intense than in gels prepared without electric-field assistance, suggesting a higher degree of supramolecular ordering and more defined intermolecular hydrogen-bonding within the assembled domains. This interpretation is consistent with the POM and TEM observations showing formation of larger anisotropic fibrils and aggregate structures under electric-field-assisted cooling. At the same time, the simultaneous presence of free NH and amide functionalities indicates that a fraction of gelator molecules remains non-associated or only weakly associated within the system. These findings demonstrate that electric field application during gel formation significantly modifies the self-assembly pathway of the oxalamide gelators.

Beyond fundamental interest, stabilization of MBBA in the nematic phase over an extended temperature range is technologically relevant, as MBBA-based systems are limited by low-temperature crystallization and by the known environmental sensitivity of Schiff-base liquid crystals toward oxygen and moisture. Notably, both gel systems remained macroscopically stable without observable crystallization during storage for at least one year under ambient conditions, further demonstrating the long-term stabilizing effect of the supramolecular gel network on the MBBA mesophase. Detailed thermal, structural, and morphological characterization of the resulting liquid-crystalline physical gels is presented in the following section.

### 2.2. Thermal and Optical Characterization (POM) of LC Gels

**Figure 1:**
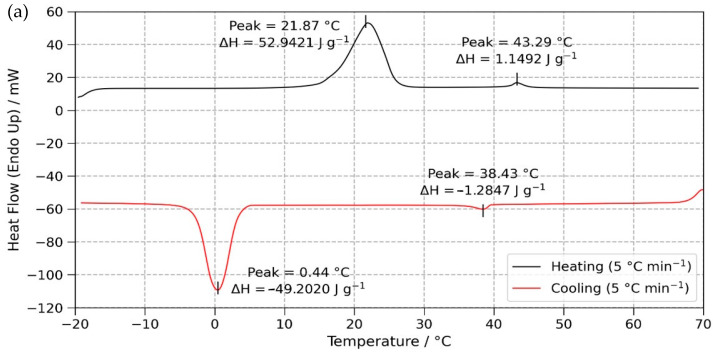

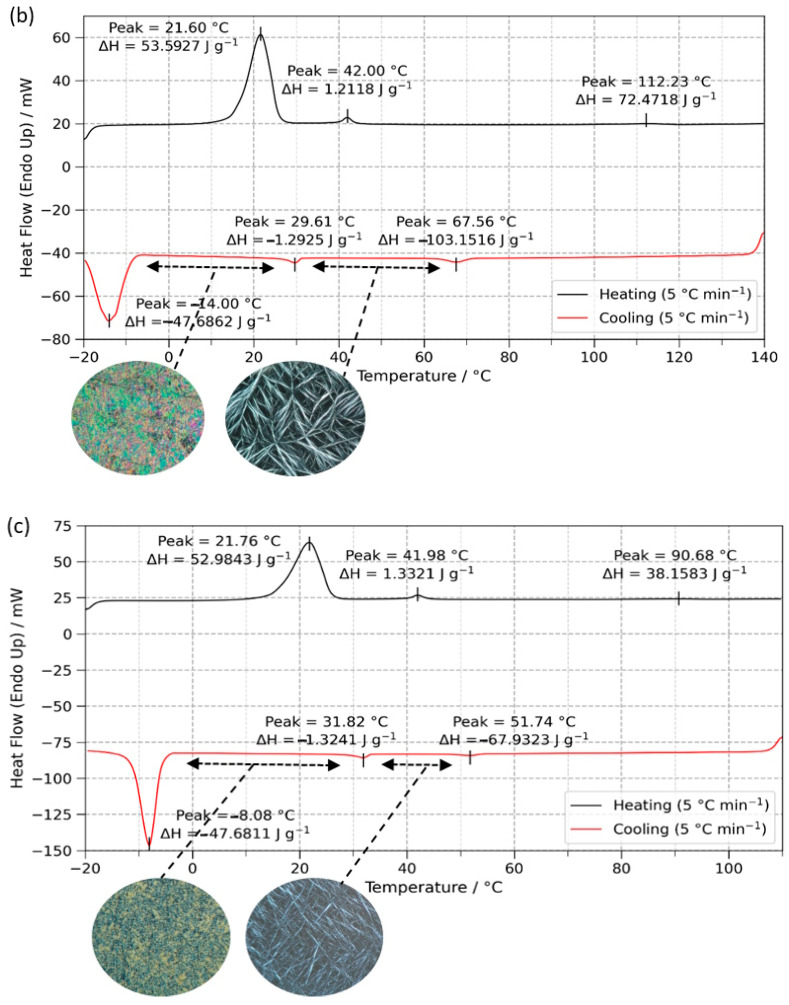
DSC thermograms of MBBA and gel samples, coupled with POM micrographs, with cooling and heating cycles at 5 °C min^−1^: (**a**) MBBA; (**b**) 6-O-Me; (**c**) 9-O-Me.

In order to explore the thermal transition behavior of the liquid crystal physical gels, DSC measurements, accompanied with optical polarizing microscopy (POM), were conducted. The DSC thermograms of MBBA and the resulting gels, coupled with POM micrographs, are presented in Figure 1.

The DSC thermograms reveal distinct phase transitions for both 6-O-Me (Figure 1b) and 9-O-Me (Figure 1c) liquid crystal physical gels.

On cooling, 6-O-Me shows gelation at 71.07 °C (peak 67.56 °C) by forming fibrous aggregates (Figure 1b and Figure S1a in the Supplementary Information). The isotropic-nematic transition, characterized by the appearance of the typical nematic droplets occurs at 31.10 °C (peak 29.61 °C), which further develop into a marble texture (Figure 1b and Figure S1b in the Supplementary Information) [23]. Further cooling results in crystallization at −14.00 °C (onset −10.41 °C). Upon heating, the crystal–nematic transition peaks at 21.60 °C, displaying a marble texture. The nematic–isotropic transition at 42.00 °C, and the sol–gel transition at 112.23 °C (broad interval 90–120 °C).

Similar behavior was observed for the 9-O-Me gel (Figure 1c and Figure S1c–e in the Supplementary Information). During cooling, gelation occurs at 54.21 °C (peak 51.74 °C), the isotropic–nematic transition at 33.18 °C (peak 31.82 °C), and crystallization at −8.08 °C. Heating reveals transitions at 21.76 °C (crystal–nematic), 41.98 °C (nematic–isotropic), and 90.68 °C (sol–gel, 75–98 °C interval). The presence of the nematic phase was confirmed by the characteristic marble textures. Additional POM micrographs associated with the thermal transitions are provided in Figure S1 (Supplementary Information).

**Figure 2:**
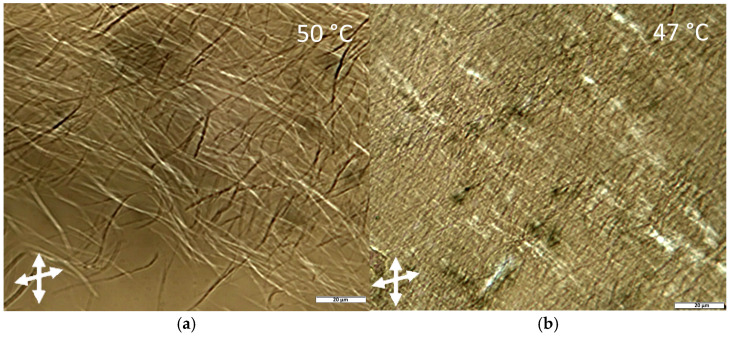
POM Micrographs of LC gels obtained on cooling from the isotropic phase: (**a**) 6-O-Me at 50 °C; (**b**) 9-O-Me at 47 °C, the white arrows present the position of the polarizers (scale bar 20 µm).

Both gelators show minimal influence on the isotropic–nematic transition of MBBA, with only a slight shift of 3–4 °C (Figure 1a–c). Their primary effect is the suppression of crystallization, delaying it from 0 °C in pure MBBA to −14 °C (6-O-Me) and −8 °C (9-O-Me). The enthalpy values remain nearly unchanged, indicating that the liquid crystal organization is preserved. The sol–gel transitions occur in the isotropic phase during cooling at 67 °C (6-O-Me) and 51 °C (9-O-Me), confirming their classification as type I liquid crystalline physical gels. Additionally, POM of both 6-O-Me and 9-O-Me liquid crystal physical gels allow direct comparison of the morphologies formed by the two structurally distinct gelators within the MBBA mesogen (Figure 2). POM images of the samples without cover glass reveal that the 6-O-Me gelator produces larger fibrous aggregates or stacked domains, while the 9-O-Me gelator forms a more uniform and finely distributed fibrous network. This indicates that spacer length exerts a significant influence on aggregate diameter and overall network organization within the liquid crystal medium.

Although the 6-O-Me gel appears sparser in POM images, it causes a larger crystallization delay compared to the observed for the 9-O-Me gelator. This enhanced stabilization is likely related to the greater thermal robustness of the 6-O-Me network, as indicated by its higher gel–melting temperature and enthalpy values.

### 2.3. TEM and SEM Microscopic Analysis of Gel Morphology

**Figure 3:**
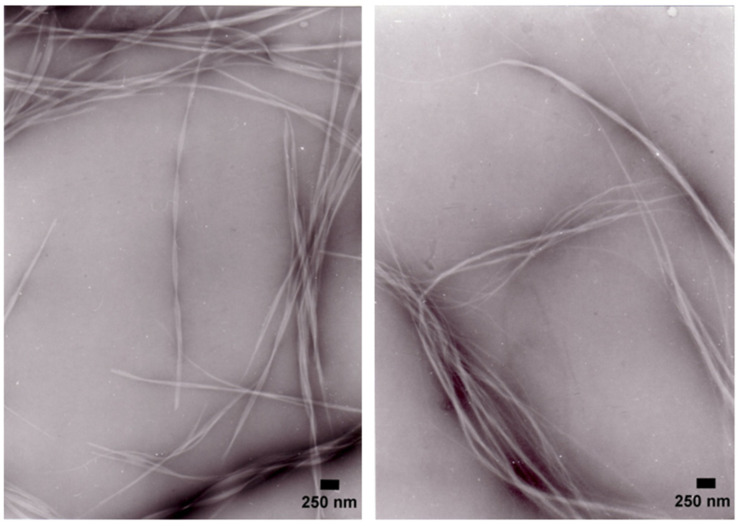
TEM micrographs of gels in the isotropic phase of MBBA for the gelators: 6-O-Me (**left**) and 9-O-Me (**right**).

Both gelators are chiral molecules, capable of forming ordered supramolecular structures through a combination of Van der Waals interactions and directional hydrogen bonding mediated by the oxalamide groups. TEM microscopic studies confirmed the formation of long fibrillar structures with diameters ranging from 30 to 60 nm (Figure 3). The 6-O-Me gel exhibits more pronounced fiber chirality than the 9-O-Me analogue, as evident in Figure 3. The difference in self-assembly behavior can be attributed to the even–odd effect of the methylene spacer: the six-carbon spacer (even) favors more twisted or helical arrangements in ribbons, whereas the nine-carbon spacer (odd) promotes the formation of more uniform, straight, and densely packed fibrils, resulting in a denser but less chiral network. These structural variations directly impact the resulting fiber morphology and packing density in the isotropic phase of the liquid crystal. The results clearly demonstrate that solvent polarity and anisotropic media significantly influence the self-assembly of the gelators compared to their organization in organic solvents of different polarities reported previously [21].

**Figure 4:**
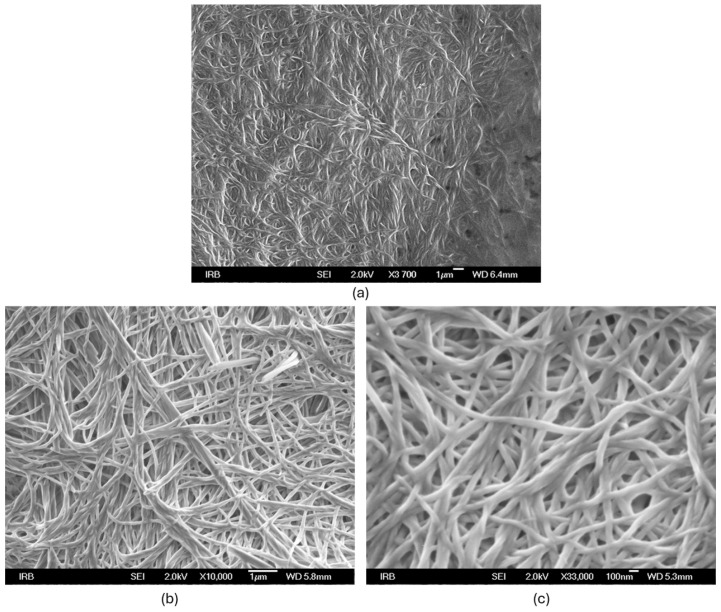
SEM micrographs of gels in the isotropic phase of MBBA: (**a**) 6-O-Me; (**b**) 6-O-Me; (**c**) 9-O-Me.

In addition to TEM, further morphological insight was obtained using scanning electron microscopy (SEM) (Figure 4). Both techniques confirmed the presence of a continuous, interconnected fibrous network homogeneously distributed within the MBBA matrix. The SEM micrographs show that both gels possess highly entangled networks that contribute to the suppression of crystallization in the nematic phase, consistent with DSC observations. This phenomenon arises from the formation of nanoscale cavities within the gel matrix that encapsulate portions of the liquid crystal, thereby stabilizing the mesophase and retarding crystallization.

Both POM and TEM analyses confirm a higher concentration of chiral aggregates in the 6-O-Me gel, which may further influence crystallization kinetics by imposing local asymmetry and hindering molecular alignment during cooling.

Together, these observations demonstrate that subtle differences in molecular structure, specifically spacer length and parity, govern both the self-assembly behavior and thermal–mechanical response of these liquid crystal physical gels.

Although the present combination of DSC, POM, TEM, SEM, rheology, electrorheology, and FTIR provides consistent evidence for the formation and organisation of supramolecular fibrillar networks in MBBA, techniques capable of resolving the detailed supramolecular packing, such as X-ray scattering or in situ spectroscopic measurements under applied electric field, were not performed in this study. Therefore, the proposed molecular-level interpretation should be regarded as being supported by combined but not fully structure-resolving evidence.

### 2.4. Rheological Characterization

**Figure 5:**
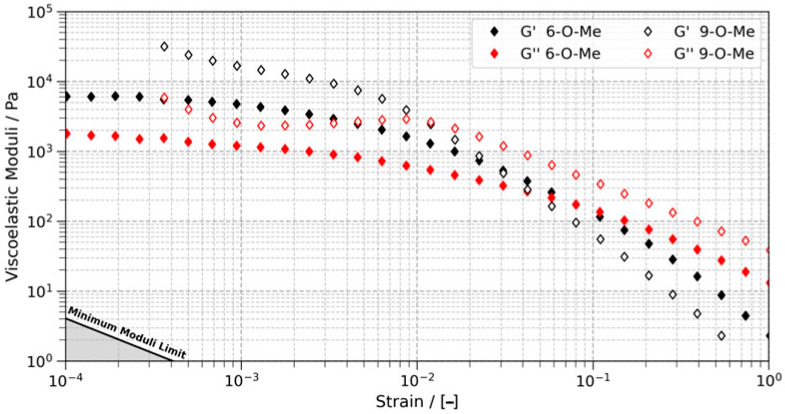
Results from the amplitude sweep experiments at 1 rad/s at 25 °C.

The viscoelastic properties of the 6-O-Me and 9-O-Me liquid crystal physical gels were characterized through small amplitude oscillatory shear experiments. From amplitude sweep tests performed at 25 °C (Figure 5), it can be observed that both systems exhibit a typical gel-like response, where the storage modulus (G′) remains higher than the loss modulus (G″) over low strain amplitudes, confirming the predominance of elastic behavior within the linear viscoelastic region (LVR). This indicates the presence of a well-defined, stable gel network at small deformations. The 9-O-Me gel demonstrates a consistently higher storage modulus compared to the 6-O-Me gel, suggesting a denser and more rigid network structure, likely resulting from stronger intermolecular interactions or enhanced packing efficiency within the self-assembled fibrillar network. The slightly higher strain onset observed for the 9-O-Me gel is attributed to unmet minimum torque requirements.

**Figure 6:**
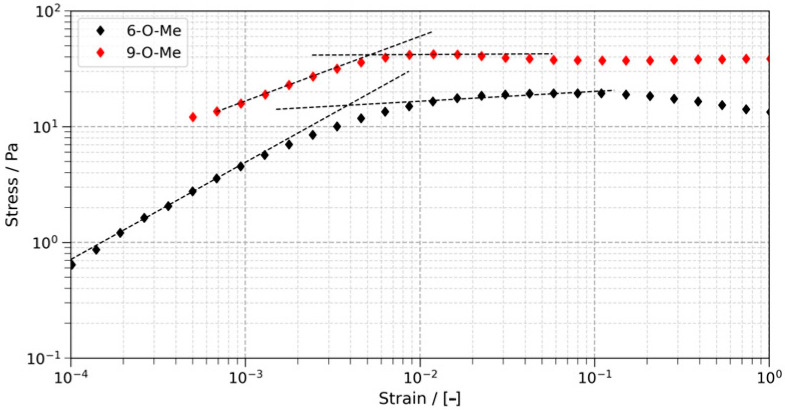
Stress as a function of strain for both gels with power law fittings for behavior at low and high strains.

The yield stress, defined as the critical stress marking the transition from elastic-dominated to flow-dominated behavior, was determined from oscillatory stress–strain data (Figure 6) using a power-law fitting approach for pre- and post-yield regions [24]. Both, the low- and high-strain regions were fitted using a power-law model:

$$\tau =K\gamma^{n},$$

where τ is the stress, γ is the strain, K is the consistency factor, and n is the power-law exponent. The fitted parameters are provided in the Supplementary Information (Table S1). Additional validation of the yield stress values was obtained from the evolution of tanδ as a function of the amplitude of the oscillatory stress (Figure S2, Supplementary Information), where the onset of the sharp increase in tanδ was found to occur at stress values consistent with those obtained from the power-law fitting analysis.

The 9-O-Me gel exhibits a yield stress of 41.71 Pa, approximately 2.7 times higher than that of the 6-O-Me gel (15.28 Pa), confirming the formation of a more robust network. Corresponding yield strain values were 0.52% for 9-O-Me and 0.39% for 6-O-Me, further supporting the increased elasticity and structural integrity of the longer-spacer gelator system. This correlation between molecular architecture and mechanical strength highlights the significant role of spacer length in modulating network stiffness and resistance to deformation.

The differences in the rheological parameters of the investigated liquid-crystalline physical gels can be mainly attributed to differences in supramolecular network density, fibrillar morphology, and aggregate organization. At identical gelator concentration, denser, more homogeneous fibrillar networks provide more efficient stress transfer throughout the gel matrix, resulting in higher storage modulus values and enhanced resistance to deformation. In this context, the higher storage modulus and yield stress observed for the 9-O-Me gel are consistent with the formation of a more cohesive, mechanically effective network, as supported by the morphological observations. Conversely, a less homogeneous or more sparsely connected network is expected to deform more easily, leading to lower elastic modulus and yield stress values.

**Figure 7:**
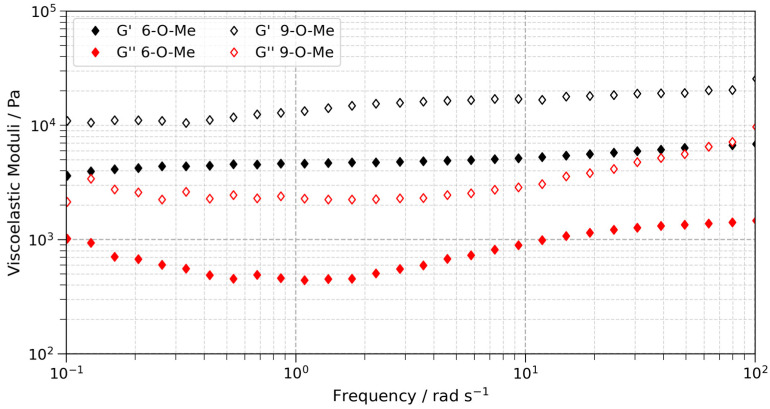
Frequency sweep of both gels at 25 °C.

To further assess the gels’ mechanical stability, frequency sweep tests were performed within the LVR (Figure 7). Both gels exhibited nearly constant moduli values across the examined frequency range, demonstrating strong structural stability at different timescales. No crossover between moduli was observed, confirming the dominance of elastic behavior and the persistence of the gel state throughout the test. The absence of an inertia-related upturn at high frequencies validates the reliability of the measurements. Overall, these results confirm that both 6-O-Me and 9-O-Me form stable, self-supporting physical gels, with the 9-O-Me gel exhibiting superior mechanical strength due to its more cohesive network structure.

### 2.5. Electrorheological Characterization

The influence of external electric stimuli on the liquid crystal physical gels was investigated by applying an electric field of 3 kV mm^−1^ either after cooling into the nematic phase or during gel network formation and subsequent gelation.

It should be noted that the present electrorheological characterization was performed at a single electric-field strength of 3 kV mm^−1^. Therefore, the threshold field for the onset of the electrorheological response could not be determined in this study. The selected field strength was used as a proof-of-concept condition to generate a measurable mechanical and morphological response within the operational limits of the available experimental setup. Consequently, the present results should not be interpreted as an optimized operating condition for practical devices.

Due to instrumental constraints associated with the electrorheological cell, specifically, the maximum allowable current output of the voltage generator, measurements are limited by the electrical conductivity of the samples [20]. As the 9-O-Me gel exhibits relatively high conductivity, only the 6-O-Me gel could be characterized under an applied field.

**Figure 8:**
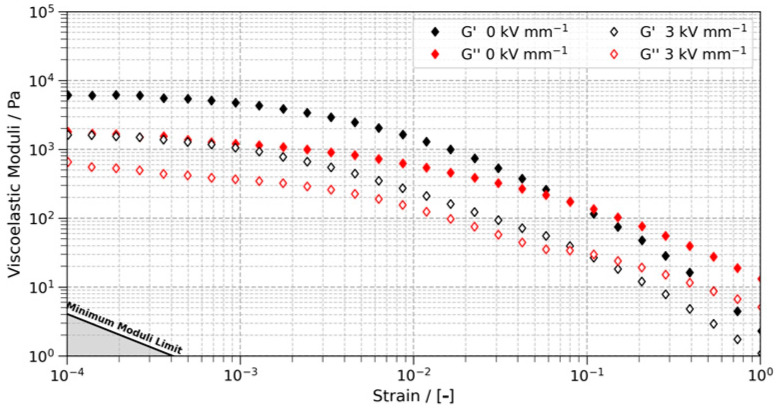
Electrorheological amplitude sweeps measurements of 6-O-Me/MBBA liquid-crystalline physical gels under two electric-field application protocols: (filled symbols) electric field applied after gel formation at room temperature; (open symbols) electric field applied during cooling from the isotropic phase through gelation.

Amplitude sweeps tests performed at 25 °C (nematic phase) with and without electric field application (Figure 8) reveal a noticeable weakening of the gel network under electrical stimulation. Both storage and loss moduli decrease upon field application, indicating a reduction in network rigidity. This effect can be attributed to electric-field-induced reorientation of MBBA mesogens within the gel network cavities. MBBA possesses negative dielectric anisotropy, leading its molecules to align perpendicular to the applied electric field. In the pre-formed gel matrix, this reorientation influences the noncovalent interactions—particularly hydrogen bonding and van der Waals forces—between the gelator molecules and the surrounding liquid crystal, thereby possibly contributing to the reduced cohesion of the supramolecular network. The electric field primarily affects the MBBA liquid crystal molecules. Their field-induced reorientation and positional alignment influence the self-assembly of gelator molecules, which associate through anisotropic growth driven by hydrogen bonding via oxalamide bridges. The interaction between the gelator and the liquid crystal plays a key role in determining the efficiency of a given gelator to form a three-dimensional network within the LC medium and to induce its solidification (gelation). Under the influence of an electric field, these gelator–liquid crystal interactions lead to the formation of somewhat softer gels. In our previous study, where the behavior of a spin probe was monitored by EPR [25], it was observed that even after LC crystallization, a fraction of the spin probe remained outside the crystallized bulk phase. This finding indicates that, although the LC and gelator represent two distinct supramolecular systems with their own phase transitions, they remain mutually interactive, with gelator aggregates influencing and interacting with LC molecules.

**Figure 9:**
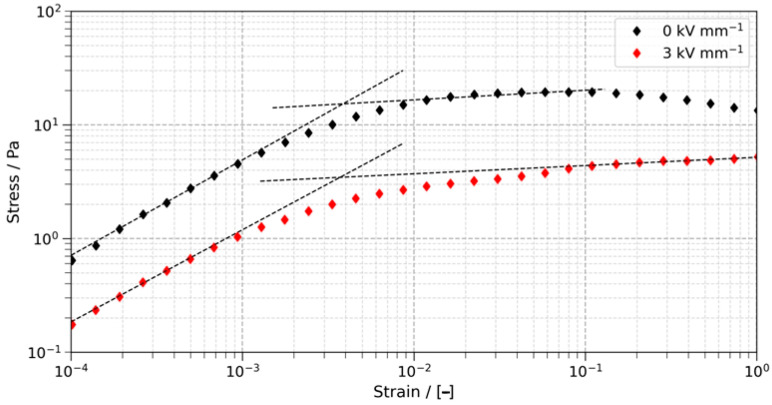
Stress as a function of strain with power law fittings for behavior at low and high strains for 6-O-Me with and without application of electric field at 25 °C.

Following the same analysis protocol as in the field-free condition, yield stress and yield strain values were extracted from the oscillatory amplitude sweep data (Figure 9). A pronounced reduction in yield stress is observed under electric field application, dropping by approximately 77%, from the baseline value to 3.45 Pa. The yield strain remains largely unchanged, suggesting that while the gel network retains its elastic threshold, its resistance to flow deformation is substantially diminished. These findings indicate that the applied electric field primarily weakens the gel’s cohesive network strength rather than altering its fundamental viscoelastic nature.

**Figure 10:**
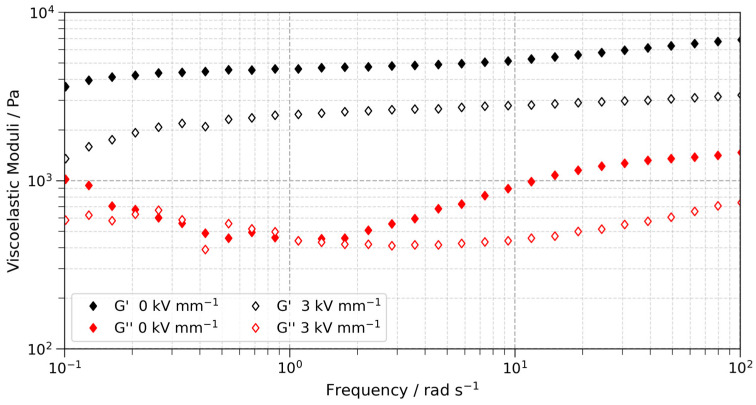
Frequency sweeps of 6-O-Me with and without application of electric field at 25 °C.

To further assess the mechanical stability of the electrically perturbed network, frequency sweep tests were conducted under identical thermal and field conditions (Figure 10). Despite the reduction in G′ compared to the field-free case, both moduli exhibit plateau behavior across the tested frequency range, with no crossover between G′ and G″. This indicates that the gel retains its essential gel-like character and remains structurally stable across short- and long-term deformation modes, even after partial weakening induced by the field.

**Figure 11:**
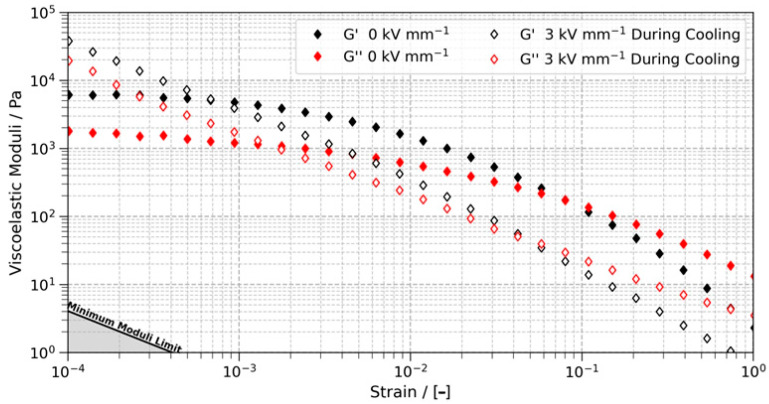
Amplitude sweeps of 6-O-Me gel with and without application of electric field during cooling at 25 °C.

The influence of the electric field during network formation was examined by applying the same field strength (3 kV mm^−1^) during the cooling process and maintaining it at 25 °C during subsequent measurements (Figure 11). Under these conditions, the gel exhibits significant structural disruption. Although G′ remains higher than G″ at low strains, indicating partial gel-like behavior, the absence of a well-defined linear viscoelastic region suggests that proper network organization was hindered during gelation. This observation implies that the electric field interferes with the self-assembly process of the gelator molecules and the liquid crystal domains, likely by inducing premature mesogen alignment or disrupting intermolecular interactions necessary for uniform fiber formation.

Overall, these results demonstrate that while the 6-O-Me gel maintains its gel-like behavior under post-formation electric field application, the field substantially weakens the network by disrupting mesogen–gelator interactions. When applied during cooling and gelation, the field has an even more pronounced effect, preventing the establishment of a cohesive and stable gel network.

### 2.6. POM and TEM Investigation of LC Gels in a Microfluidic Setup

To visualize the effects observed in the electrorheological tests of the 6-O-Me gel, and to overcome the instrumental limitations that prevented characterization of the 9-O-Me sample, a custom microfluidic device capable of applying controlled electric fields during microscopic observation was developed. This setup enables in situ visualization of field-induced phenomena in liquid crystal physical gels under well-defined geometrical and electrical conditions. A solution of the gelator dissolved in the isotropic liquid crystal (LC) phase at 130 °C was introduced into a microfluidic setup to investigate the influence of an electric field on self-assembly and gel formation. This experimental design allows the simultaneous evaluation of two key factors: (i) the effect of confined space, which plays an important role in gelator behavior within the narrow microfluidic channels, and (ii) a well-defined regime of electric field application. TEM analysis of 6-O-Me LC gels prepared under these conditions reveals distinct differences depending on the timing of electric field application. Samples were obtained either by applying the electric field after gel formation (RT) or throughout the entire cooling process of the isotropic solution, prior to gelator aggregation (marked all-cooling sample). In both cases, the influence of confinement is evident, as gelation within the restricted geometry leads to the formation of partially oriented gelator fibers.

**Figure 12:**
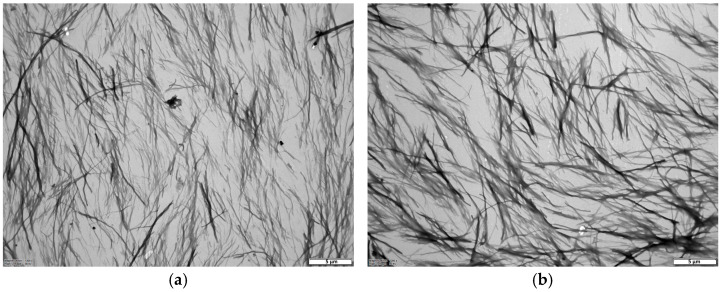
TEM images of 6-O-Me/MBBA LC gels prepared in microfluidic channels under the influence of an electric field: (**a**) applied after gel formation at RT, and (**b**) applied during cooling from the isotropic state through the gelation process to RT.

The TEM images, obtained at the same magnification, indicate clear differences in the network morphology. The gel network formed under continuous electric field application during cooling consists of significantly larger aggregates, appearing as bundles of ribbons (Figure 12b). Such structures are likely associated with reduced gelation efficiency and lower elasticity, consistent with the results obtained from electrorheological measurements. In sample (a), obtained under an electric field applied at RT, the network consists of oriented fibers and ribbon-like structures predominantly in the range of 50–100 nm in diameter. Larger aggregates are also present, with sizes ranging from 200 to 500 nm. The network appears relatively dense and homogeneously distributed fibers (Figure 12a). In contrast, sample 6-O-Me/MBBA (Figure 12b), formed under continuous electric field application during cooling, shows a reduced fraction of fiber diameters 50–100 nm and a more pronounced presence of larger aggregates, in the range of 300–600 nm. The network is less homogeneous, with increased aggregation into bundled structures.

The stronger structural changes observed when the electric field is applied continuously during cooling, compared with application to an already formed gel at room temperature, suggest that the field primarily influences the self-assembly pathway during nucleation and aggregate growth rather than substantially reorganizing a pre-existing fibrillar network. This interpretation is consistent with the FTIR results, where the field-assisted cooling sample exhibited simultaneous presence of hydrogen-bonded and free oxalamide functionalities together with sharper amide bands, indicating modified supramolecular organization during assembly (Supplementary Information Figure S8). At present, it remains unclear whether this effect originates from direct interaction of the electric field with the polar oxalamide functionalities or indirectly through field-induced ordering of MBBA mesogens, which may provide an anisotropically organized template for aggregate growth.

**Figure 13:**
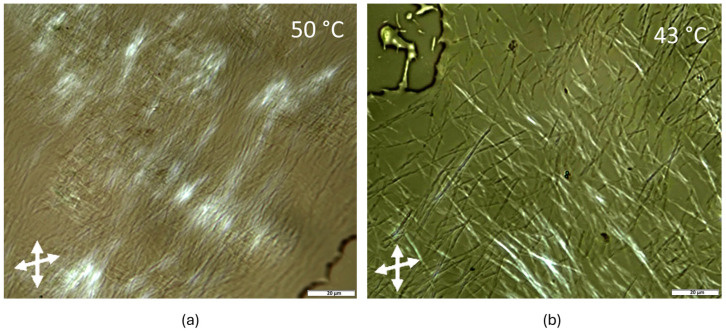
POM images of an uncovered sample of 6-O-Me/MBBA LC gels obtained on heating, prepared in microfluidic channels under the influence of an electric field: (**a**) applied after gel formation at RT, and (**b**) applied during cooling from the isotropic state through the gelation process to RT, the white arrows present the position of the polarizers (scale bar 20 µm).

Further analysis by polarized optical microscopy confirms these observations, revealing the formation of thicker, more oriented fibers in samples prepared under continuous electric field application during cooling process (Figure 13). In contrast, samples exposed to the electric field after gel formation at room temperature (RT) exhibit a more homogeneous and directional fibers network within the microfluidic setup.

Although the application of an electric field during gelation enhances fiber robustness, it also introduces reduced overall structural order, yielding a network that deviates from the homogeneous and randomly distributed morphology typically formed under field-free conditions. These findings provide direct visual evidence supporting the electrorheological behavior previously described for the 6-O-Me gel, illustrating how external electric fields modulate both the microstructure and mesogenic organization within the liquid crystal gel matrix.

Upon reheating and subsequent cooling of the samples on a glass slide, an isotropically homogeneous network is re-formed, indicating the loss of structural memory induced by both confined space and the electric field (Supplementary Information Figure S7).

The proposed mechanism of field-induced network weakening is therefore based on the combined evidence from electrorheological measurements and microscopic and spectroscopic observations. The reduction in storage modulus and yield stress, together with the formation of larger and more oriented aggregates under electric-field-assisted cooling, is consistent with electric-field-induced reorganisation of the MBBA mesogens and the consequent perturbation of gelator self-assembly. FTIR spectroscopy provides additional evidence for hydrogen-bonded oxalamide assemblies in the MBBA gels and indicates that electric-field-assisted cooling modifies the association state and supramolecular ordering of the gelator network. However, the present data do not allow us to unambiguously determine whether the field acts directly on the oxalamide hydrogen-bonding motif or indirectly through field-induced reorientation of MBBA mesogens, thereby altering the aggregation pathway of the gelator. Considering the known electric-field responsiveness of MBBA, the latter mechanism appears more likely, although further in situ molecular-level studies would be required to confirm it.

Overall, these experiments demonstrate that gelator self-assembly can be effectively modulated by external factors, including confinement and electric fields, enabling control over aggregate formation and, consequently, over the macroscopic properties of dual-responsive systems.

## 3. Conclusions

This study provides a detailed investigation of 6-O-Me and 9-O-Me liquid crystal physical gels (LCPG) in MBBA, focusing on their thermal, mechanical, and electromechanical behavior. Both gelators successfully form self-assembled fibrous networks, which impart solid-like mechanical characteristics while maintaining the intrinsic nematic order of the liquid crystal host.

Thermal analysis revealed that while the gelators have minimal impact on the isotropic–nematic transition, they significantly suppress crystallization, delaying it from 0 °C to −8 °C for 9-O-Me and to −14 °C for 6-O-Me. This crystallization delay highlights the ability of the gel network to stabilize the nematic phase at lower temperatures, which is particularly important for applications that require reliable liquid crystal performance in cold environments. The stabilization is attributed to cavities formed within the gel network, which trap and organize the liquid crystal molecules. The chiral 6-O-Me gelator exhibits a stronger effect, promoting extended nematic stability through enhanced fiber chirality observed at the microscopic level.

Rheological measurements confirmed that both gels exhibit gel-like behavior, with the storage modulus (G′) exceeding the loss modulus (G″) at low strains. Networks remained mechanically stable under both short- and long-term deformations. The 9-O-Me gel displayed higher elastic modulus and yield stress than the 6-O-Me gel, consistent with a denser and more cohesive network. Electrorheological tests showed that electric fields weaken the 6-O-Me gel network in the nematic phase and significantly influence network formation during cooling, producing thicker, anisotropic fiber aggregates. These effects were corroborated by TEM and polarized optical microscopy using a microfluidic device, providing direct visualization of field-induced alignment and structural reorganization.

Overall, this work demonstrates that LCPGs combine the mechanical robustness of soft solids with the stimuli-responsiveness of liquid crystals, and that their ability to delay crystallization at low temperatures is a key feature for practical applications. The viscoelastic and structural properties can be tuned through gelator structure and external electric fields, supporting their potential in adaptive optics, soft actuators, and low-temperature responsive devices.

A detailed mechanistic distinction between direct electric-field interaction with the polar oxalamide functionalities and indirect liquid-crystal-mediated templating effects remains beyond the scope of the present study and will require systematic investigation across different temperatures, host media, and field conditions.

Some limitations should nevertheless be considered when interpreting these results. The electrorheological response was examined at a single electric-field strength of 3 kV mm^−1^, preventing determination of the threshold field and optimized operating conditions. Future work should systematically assess the effects of field strength, temperature, gelator concentration, gelator molecular structure, cell gap, and liquid-crystal host composition to clarify the molecular mechanism and enable low-voltage optimization. This is particularly relevant because comparable electric-field strengths may be achieved at substantially lower absolute voltages in thin-film or microstructured electro-responsive systems.

## 4. Materials and Methods

### 4.1. Materials

The nematic liquid crystal MBBA (PT: Cr 22 N 44 Iso [25]) was sourced from BLD Pharm (99.53% purity) and used without further modifications.

### 4.2. Organic Gelators

The synthesis of the compounds 6-bis((O-leucylmethanol)-N-yloxalamido)hexane (6-O-Me) and 1,9-bis((O-leucylmethanol)-N-yloxalamido)nonane (9-O-Me) have been prepared according to the procedure described elsewhere [21]. The synthetic procedure is described in the Supplementary Information.

### 4.3. Preparation of LC Physical Gels

The liquid crystal physical gels were prepared by dissolving both gelators (1.6 wt.%) in MBBA at 150 °C in clear vials. After total dissolution was achieved, the solution was cooled down to ambient conditions. Each resulting gel was named after the gelator used to prepare it, namely 6-O-Me and 9-O-Me.

### 4.4. Characterization

Rheological properties of 6-O-Me and 9-O-Me liquid crystal physical gels were measured using a stress-controlled rheometer (MCR 301, Anton Paar GmbH, Graz, Austria) equipped with parallel-plate geometry (50 mm diameter). A 0.5 mm gap was maintained for all samples. Gels were heated above their melting temperatures (6-O-Me: 150 °C; 9-O-Me: 120 °C), transferred to the rheometer, and cooled at a rate of 1 °C min^−1^ to the test temperature, thereby inducing gelation. Strain sweep tests were performed at 25 °C (1 rad s^−1^, strain range 0.0001–1) to determine the linear viscoelastic region and frequency sweeps (0.1–100 rad s^−1^) were conducted to evaluate viscoelastic behavior. Yield stress was determined from the intersection of low- and high-strain power-law fits as described elsewhere [26].

Electrorheological properties of the gels were analyzed using the same rheometer setup equipped with an electrorheological cell, allowing the application of an external electric field perpendicularly oriented to the shear flow, as in our previous work [20].

Two testing protocols were employed: (i) field application during cooling and measurement, and (ii) field application during measurement following field-free cooling. A constant field strength of 3 kV mm^−1^ was maintained using a high-voltage supply (HCL 14-12500, FuG Elektronik GmbH, Schechen, Germany). The 9-O-Me gel’s high electrical conductivity rendered the electrorheological characterization impracticable under the current experimental setup, due to the maximum current output of the available high-voltage supply. For 6-O-Me, a constant electric field strength of 3 kV mm^−1^ was maintained throughout testing, though temperature-dependent conductivity variations restricted full field application for temperatures above 60 °C. All measurements were performed under small-amplitude oscillatory shear (SAOS) conditions consistent with field-free reference tests.

For both rheological and electrorheological characterization, the reliable experimental windows for accurate data interpretation were defined and plotted as detailed in the seminal work of Ewoldt et al. [27].

Thermal transitions of the gels were characterized using a DSC instrument at controlled heating and cooling rates of 5°C min^−1^. DSC was carried out on a Perkin-Elmer Diamond DSC (PerkinElmer, Waltham, MA, USA). For measurements on the gels, a weighed amount of gelator and a weighed amount of sample were placed in a 60 µL stainless steel sample cup, which was sealed directly. The sample cup was placed in the DSC apparatus together with an empty sample cup as a reference. Heating and cooling scans were recorded at scan rates of 2 and 5 °C min^−1^. Repeated heating and cooling of the samples were highly reproducible, and prolonged ageing times did not affect the results.

Optical textures were determined using an Olympus BX51 polarizing microscope, Tokyo, Japan equipped with a Linkam TH600 hot stage Surrey, UK, a PR600 temperature controller, and an EP50 Olympus camera. The samples were observed on plain glass slides without the cover. The samples of 6-O-Me-all cooling, 6-O-Me-RT, 9-O-Me-all cooling, and 9-O-Me-RT were taken from the microfluidic channels, placed on untreated glass slides without a cover, and heated at a rate of 5 °C/min. Micrographs were recorded at selected temperatures to monitor phase transitions and gel morphology. Transmission electron microscopy (TEM) and scanning electron microscope (SEM) analysis provided further insights into fiber morphology.

TEM analysis was performed using a JEOL JEM-1400Flash HC transmission electron microscope (JEOL Ltd., Tokyo, Japan), operated at an accelerating voltage of 60–120 kV. For electron microscopy a piece of gel was placed on a copper grid and removed after 20 s leaving some patches of the gel on the grid. The gel sample was examined by SEM using a JEOL JSM-7000F field-emission scanning electron microscope (JEOL, Japan).

**Figure 14:**
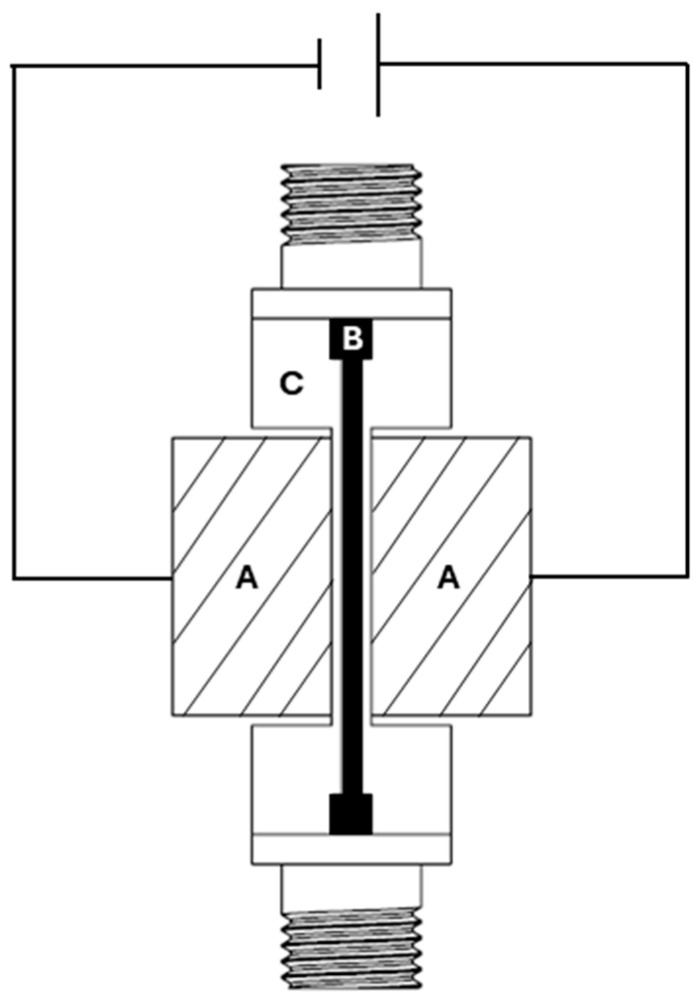
Microfluidic device with electric field application: (A) aluminum inserts connected to power source; (B) flow channel; (C) 3D resin-printed device.

An electro-microfluidic device, represented in Figure 14, was designed and fabricated (Supplementary Information Figures S3–S6) by resin 3D printing (Elegoo Mars 5 Ultra Shenzhen, China, 0.05 mm layer height) using Phrozen TR300 resin Hsinchu, Taiwan (HDT 160 °C). Post-curing was performed in Form Cure V1 at 60 °C and 405 nm light for 30 min. Glass slides and aluminum inserts were attached with a refractory glue, and the microchannel was sealed with resin-printed caps. Gel samples were injected using a glass syringe and subjected to electric field tests under two conditions: in situ field application during cooling and post-cooling field application at ambient temperature. Electric fields (3 kV mm^−1^) were supplied by a LabSmith HVS448 sequencer, Livermore, CA, USA. Device dimensions and print quality were analyzed using an inverted microscope (Leica DM IL LED) equipped with a CCD camera (DFC350 FX, Leica, Wetzlar, Germany) at 2.5× magnification, with further information in Supplementary Information.

## Data Availability

The data that support the findings of this study are available from the corresponding author upon reasonable request.

## Supplementary Materials

The following supporting information can be downloaded at: https://www.mdpi.com/article/10.3390/gels12060485/s1.
