# Supplementary material for: Electric-Field-Induced Modulation of Structure and Rheology in MBBA-Based Liquid Crystal Physical Gels
Source: Gels. 2026 Jun 1;12(6):485. doi: 10.3390/gels12060485 (PMC13297764; doi:10.3390/gels12060485)
Supplement: Supplementary file 1 [file gels-12-00485-s001.zip › gels-4279506-supplementary.pdf]

Supplementary Information

For

Electric-Field-Induced Modulation of Structure and Rheology in MBBA-Based Liquid Crystal Physical Gels

André Cruz [1], Andreja Lesac [2], Nataša Šijaković Vujičić [2,*] and Francisco J. Galindo-Rosales [1,*]

1 Transport Phenomena Research Center (CEFT), Associate Laboratory in Chemical Engineering (ALiCE), Department of Chemical and Biological Engineering, Faculty of Engineering, University of Porto, Rua Dr. Roberto Frias, 4200-465 Porto, Portugal
2 Division of Organic Chemistry and Biochemistry, Ruđer Bošković Institute, Bijenička c. 54, 10000 Zagreb, Croatia; alesac@irb.hr
* Correspondence: nsijakov@irb.hr (N.Š.V.); galindo@fe.up.pt (F.J.G.-R.)

Synthesis of the gelators

Melting points were determined on a Kofler stage and are uncorrected. Optical rotations were measured on an optical activity AA-10 automatic polarimeter at 589.3 nm. The one- and two-dimensional homo- and heteronuclear ^{1}H and ^{13}C NMR spectra were recorded with Bruker AV-300 and Bruker AV-600 spectrometers, operating at 300 and 600 MHz for the ^{1}H nucleus and 75 and 150 MHz for the ^{13}C nucleus, respectively. FTIR spectra were recorded on a Bomen MB 102 spectrometer. Thin-layer chromatography (TLC) was performed on silica-gel-coated Merck 60 F_{254} silica plates, and were visualized using a UV lamp (254 nm) or I_2 vapours. All chemicals were of the best commercially available grade and were used without purification. Solvents were purified according to literature methods and stored over molecular sieves. Mass spectra were recorded on an Extrell FTMS 2001-DD Fourier Transform Mass Spectrometer, electron impact, ionising voltage 70 eV and 4800 MALDI TOF/TOF Analyzer, Applied Biosystems.

1,6-bis(ethoxyoxalamido)hexane (1a): A solution of diaminohexane (2.077 g, 17.87 mmol) in dry CH_2Cl_2 (30 mL) and TEA (4.38 ml, 39.32 mmol) were simultaneously added dropwise within 30 minutes to a cooled (-10 °C) solution of the ethyl oxalylchloride (4.38 ml, 39.32 mmol) in dry CH_2Cl_2 (50 ml). The stirring was continued for 30 min at 0°C, and followed by an overnight at room temperature. The CH_2Cl_2 (50 ml) was added, and the mixture was washed with water, 1.5% AcOH, 5% $NaHCO_3$ and again with water. The organic layer was dried (Na_2SO_4) and the solvent evaporated. Recrystallisation (CH_2Cl_2 / light petroleum) gives the title compound (3.673 g, 65 %). FTIR (KBr) $\tilde{\upsilon}$: 3322, 1747, 1733, 1681, 1544 cm^{-1}. ^{1}H NMR (300 MHz, $CDCl_3$, 25 °C): δ = 7.58 (bs, 2H, $CONH_{(bridge)}$), 4.20 (q, 4H, J = 7.14, $OCH_{2(ethyl)}$), 3,20 - 3,16 (m, 4H, $CH_{2(1)}$), 1,44 - 1,42 (m, 4H, $CH_{2(2)}$), 1.29-1.17 (m, 10H, $CH_{3(ethyl)}$, $CH_{2(3)}$) ppm. ^{13}C NMR (75.5 MHz, $CDCl_3$, 25 °C): δ = 160.2 (CO_{ester}); 156.5 (CONH), 62.7 ($OCH_{2(ethyl)}$), 39.2 ($CH_{2(1)}$), 28.4 ($CH_{2(2)}$), 25.8 ($CH_{2(3)}$), 13.4 ($CH_{3(ethyl)}$) ppm.

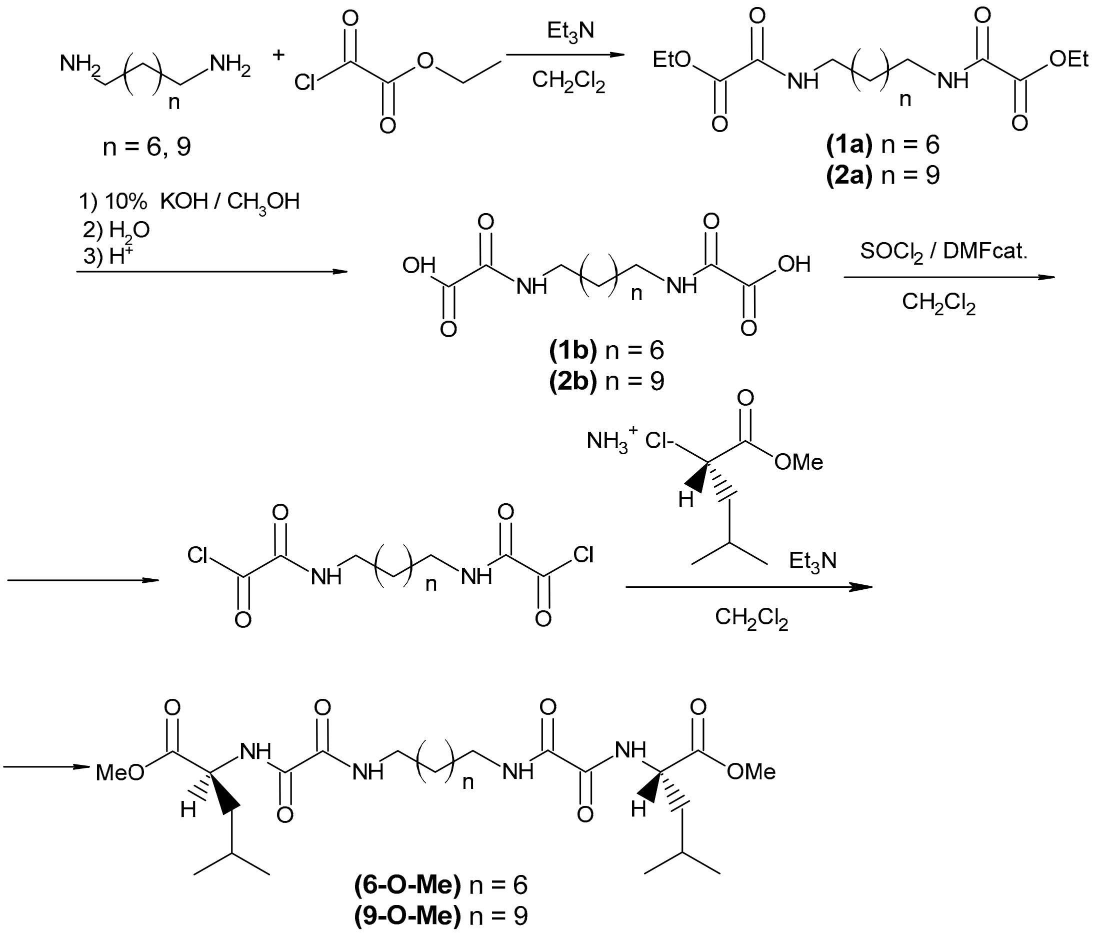

Scheme S1. Schematic representation of the synthesis of 6-O-Me and 9-O-Me gelators.

1,6-bis(hydroxyoxalamido)hexane (1b): A solution of **1a** (3.300 g, 10.43 mmol) was dissolved in 10% KOH / CH_3OH (134 mL). The reaction mixture was stirred overnight at room temperature. The methanol was evaporated, water was added, and the solution was acidified with 1M HCl until pH=2, when precipitation occurred. The precipitate was filtered off, washed with water and methanol, and dried under reduced pressure. Yield: 1.813 g, 67 %. FTIR (KBr) $\tilde{\upsilon}$: 3354, 3204, 1761, 1682, 1560 cm^{-1}. ^{1}H NMR (300 MHz, CD_3OD, 25 °C): δ = 8.87 (m, 2H, $CONH_{bridge}$), 3.35 - 3.32 (m, 4H, $CH_{2(1)}$), 1.65 - 1.60 (m, 4H, $CH_{2(2)}$), 1.46 – 1.41 (m, 4H, $CH_{2(3)}$) ppm. ^{13}C NMR (75.5 MHz, CD_3OD, 25 °C): δ = 162.9 (COOH), 160.2 (CONH), 40.8 ($CH_{2(1)}$), 30.1 ($CH_{2(2)}$), 27.6 ($CH_{2(3)}$) ppm.

1,6-bis((O-leucylmethanol)-N-yloxalamido)hexane (6-O-Me): A solution of $SOCl_2$ (5.57 mL, 76.8 mmol) and catalytic DMF (4 drops) were simultaneously added dropwise within 30 minutes to a cooled (-10 °C) solution of **1b** (1.00 g, 3.84 mmol) in dry CH_2Cl_2 (60 mL). The stirring was continued for 2 hours under reflux at 40°C. The solvent and residual HCl were evaporated under reduced pressure. This product was dissolved in dry CH_2Cl_2 (80 ml) and was simultaneously added dropwise within 30 minutes to a cooled (-10 °C) solution of H-Leu-OMe·HCl (1.536 g, 8.45 mmol) and TEA (2.25 mL, 16.14 mmol) in dry CH_2Cl_2 (40 ml). The stirring was continued overnight at room temperature. The CH_2Cl_2 (50 ml) was added, and the mixture was washed with water, 1.5% AcOH, 5% $NaHCO_3$ and again with water. The organic layer was dried (Na_2SO_4) and

the solvent evaporated. Recrystallisation (CH_2Cl_2 / *n*-hexane) gives the title compound (1.213 g, 61 %). M. p. 180 °C. $[\alpha]_D$ = -26 (c=1 in CH_2Cl_2). ^{1}H NMR (300 MHz, $CDCl_3$, 25 °C): δ = 7.90 (2H, d, *J* = 9.1, NH_{Leu}), 7.64 (m, 2H, NH_{bridge}), 4.61 – 4.54 (m, 2H, $CH_{(\alpha,Leu)}$), 3.72 (s, 6H, $OCH_{3(ester)}$), 3.32 – 3.27 (m, 4H, $CH_{2(1)}$), 1.71 – 1.61 (m, 6H, $CH_{2(\beta,Leu)}$, $CH_{(\gamma,Leu)}$), 1.60 – 1.50 (m, 4H, $CH_{2(2)}$), 1.35 (m, 4H, $CH_{2(3)}$), 0.94 – 0.92 (m, 12H, $CH_{3(\delta,Leu)}$) ppm. ^{13}C NMR (75.5 MHz, $CDCl_3$, 25 °C): δ = 171.8 (COO_{ester}), 159.6 ($CONH_{Leu}$), 159.1 ($CONH_{bridge}$), 52.3 ($OCH_{3(ester)}$), 50.9 ($CH_{(\alpha,Leu)}$), 40.9 ($CH_{2(1)}$), 39.3 ($CH_{2(\beta,Leu)}$), 28.9 ($CH_{2(2)}$), 26.1 ($CH_{2(3)}$), 24.6 ($CH_{(\gamma,Leu)}$), 22.6, 21.5 ($CH_{3(\delta,Leu)}$) ppm. FTIR (KBr) $\tilde{\upsilon}$: 3295, 1741, 1656, 1524 cm^{-1}. EA calcd. for (%) $C_{24}H_{42}N_4O_8$ (517.64): C 56.01, H 8.23, N 10.89; found: C 56.25, H 7.93, N 10.86.

diethyl 2,2'-(nonane-1,9-diylbis(azanediyl))bis(2-oxoacetate)(2a)

A solution of diaminononane (0.654 g, 4.13 mmol) in dry CH_2Cl_2 (30 mL) and TEA (1.21 ml, 8.68 mmol) were simultaneously added dropwise within 30 minutes to a cooled (-10 °C) solution of the ethyl oxalylchloride (0.97 ml, 8.68 mmol) in dry CH_2Cl_2 (50 ml). The stirring was continued for 30 min at 0°C, and followed by overnight stirring at room temperature. The CH_2Cl_2 (50 ml) was added, and the mixture was washed with water, 1.5% AcOH, 5% $NaHCO_3$ and again with water. The organic layer was dried (Na_2SO_4) and the solvent evaporated. Recrystallisation (CH_2Cl_2 / light petroleum) gave **2a** (0.903 g, 61 %). ^{1}H NMR (d_6 - DMSO) δ/ppm: 1.21-1.25 (m, 16 H, $CH_{3(ethyl)}$, $CH_{2(5)}$, $CH_{2(4)}$, $CH_{2(3)}$), 1.41 (bs, 4H, $CH_{2(2)}$), 3.08 (m, 4H, $CH_{2(1)}$), 4.19 (q, 4H, *J* = 6.99 Hz, $OCH_{2(ethyl)}$), 8.83 (bs, 2H, $CONH_{linker}$); ^{13}C NMR (d_6 - DMSO) δ/ppm: 13.9 ($CH_{3(ethyl)}$, 26.4 ($CH_{2(5)}$), 28.6 ($CH_{2(4)}$), 28.7 ($CH_{2(3)}$), 28.9 ($CH_{2(2)}$), 40.0 ($CH_{2(1)}$), 61.9 ($OCH_{2\ (ethyl)}$), 157.1 (CONH), 160.9 (COO_{ester}).

2,2'-(nonane-1,9-diylbis(azanediyl))bis(2-oxoacetic acid) (2b)

A solution of **2a** (0.835 g, 2.33 mmol) was dissolved in 10% KOH / CH_3OH (134 mL). The reaction mixture was stirred overnight at room temperature. The methanol was evaporated, water added and solution acidified with 1M HCl until pH 2 when precipitation occurred. The precipitate was filtered off, washed with water and methanol, dried under reduced pressure. Yield: (0.600 g, 86 %). ^{1}H NMR (d_6 - DMSO) δ/ppm: 1.20 (bs, 10H, $CH_{2(5)}$, $CH_{2(4)}$, $CH_{2(3)}$), 1.40 (m, 4H, $CH_{2(2)}$), 3.09 (m, 4H, $CH_{2(1)}$), 8.80 (t, 2H, *J* = 5.65 Hz, $CONH_{linker}$); ^{13}C NMR (d_6 - DMSO) δ/ppm: 26.4 ($CH_{2(5)}$), 28.7 ($CH_{2(4)}$), 28.8 ($CH_{2(3)}$), 29.0 ($CH_{2(2)}$), 40.0 ($CH_{2(1)}$), 158.3 (CONH), 162.4 (COOH).

1,9-bis((O-leucylmethanol)-N-yloxalamido)nonane (9-O-Me)

A solution of $SOCl_2$ (2.7 ml, 36.72 mmol) was added dropwise within 30 minutes to a cooled (-10 °C) solution of **2b** (0.555 g, 1.86 mmol) in dry CH_2Cl_2 (20 ml) containing catalytic amount of DMF (4 drops). The stirring was continued for 2 hours under reflux at 40°C. The solvent and residual HCl were evaporated under reduced pressure. The remaining product was dissolved in dry CH_2Cl_2 (80 ml) and simultaneously added dropwise within 30 minutes to a cooled (-10 °C) solution of H-Leu-OMe·HCl (0.743 g, 4.092 mmol) and TEA (1.09 ml, 7.81 mmol) in CH_2Cl_2 (50 ml). The stirring was continued overnight at room temperature. The CH_2Cl_2 (50 ml) was added

and the mixture washed with water, 1.5% AcOH, 5% $NaHCO_3$ and again with water. Organic layer was dried (Na_2SO_4) and the solvent evaporated. Recrystallisation (CH_2Cl_2 / *n*-hexane) give the title compound: (0.550 g, 53 %); m.p. =163-165 °C; $[\alpha]_D$ = -10 (c=1 in CH_2Cl_2); IR (KBr) ν_{max}/cm^{-1}: 3295 (NH), 1746 (COOMe), 1656 (amide I), 1523 (amide II); ^1H NMR (d_6 - DMSO) δ/ppm: 0.93 (d, 12 H, *J* = 5.5 Hz, $CH_{3(\delta,Leu)}$), 1.29 (bs, 10H, $CH_{2(3)}$,$CH_{2(4)}$, $CH_{2(5)}$), 1.55 (m, 4H, $CH_{2(2)}$), 1.68 (m, 6H, $CH_{2(\beta,Leu)}$ $CH_{(\gamma,Leu)}$), 3.30 (m, 4H, $CH_{2(1)}$), 3.73 (s, 6H, $CH_{3(OMe)}$) 4.58 (m, 2H, $CH_{(\alpha,Leu)}$), 7.57 (m, 2H, NH_{linker}), 7.87 (d, 2H, *J* =8.79 Hz, NH_{Leu}); ^{13}C NMR (CD_3OD) δ/ppm: 21.5, 22.6 ($CH_{3(\delta,Leu)}$), 24.6 ($CH_{(\gamma,Leu)}$), 26.5 ($CH_{2(3)}$), 28.8, 28.9, 28.9 ($CH_{2(2)}$, $CH_{2(3)}$,$CH_{2(4)}$), 39.5 ($CH_{2(1)}$), 41.0 ($CH_{2(\beta,Leu)}$), 50.9 ($CH_{(\alpha,Leu)}$), 52.3 ($CH_{3(OMe)}$), 159.0 ($CONH_{linker}$), 159.6 ($CONH_{Leu}$), 171.9 (COO_{ester}); Anal. Calcd. for $C_{27}H_{48}N_4O_8$ (Mr = 556.69): C 58.25, H 8.69, N 10.07 %; found: C 58.52, H 8.57, N 10.13 %.

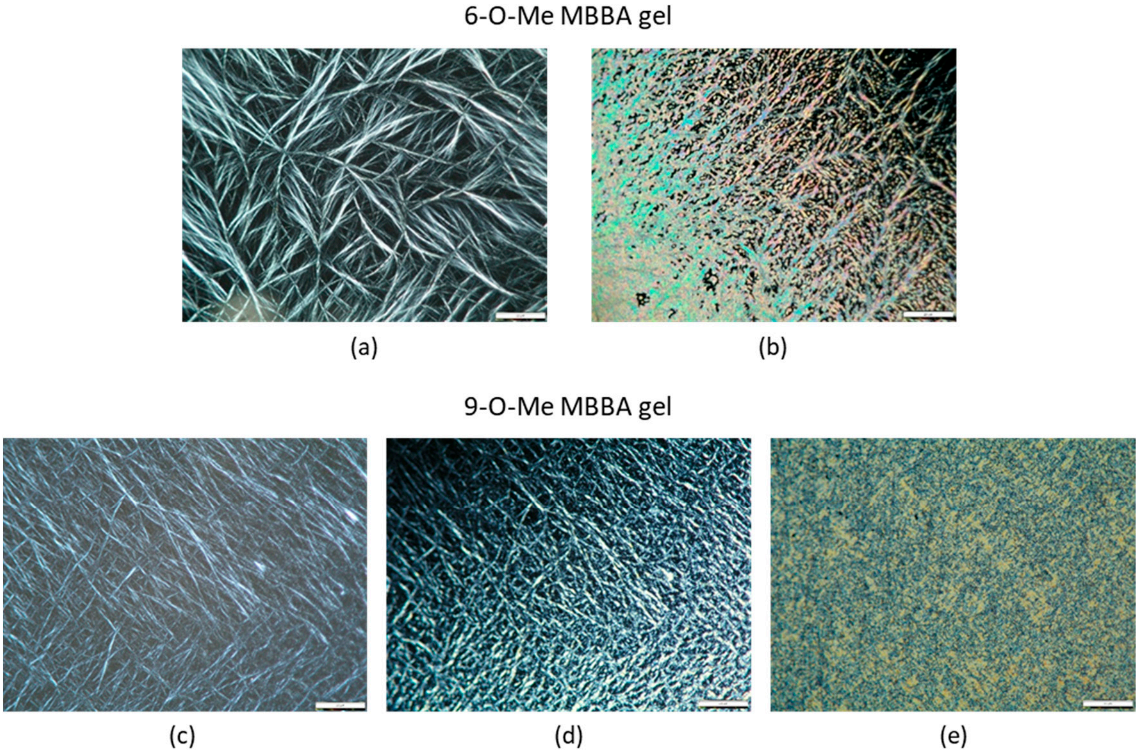

Figure S1. POM micrographs obtained on cooling (a) fibrous network of 6-O-Me MBBA gel at 32 °C; (b) texture of the nematic droplets of 6-O-Me MBBA gel at 31 °C characterizing the isotropic-nematic transition; (c) fibrous network of 9-O-Me MBBA gel at 44 °C; (d) texture of the nematic droplets of 9-O-Me MBBA gel at 32 °C characterizing the isotropic-nematic transition; (e) marble texture of the nematic phase within the gel network of 9-O-Me MBBA gel at 31 °C (scale bar 20 μm).

Table S1. Fitting parameters of the power-law equation $\tau = K\gamma^n$. where τ is the stress, γ is the strain, K is the consistency factor, and n is the power-law exponent. That equation fitted the experimental data sets obtained at low-strain and high-strain regimes. The yield stress was determined from the intersection of both.

Sample	Regime	K	n
6-O-Me (No EF)	Low strain	1621.67	0.840
6-O-Me (No EF)	High strain	24.47	0.085
9-O-Me (No EF)	Low strain	788.34	0.559
9-O-Me (No EF)	High strain	24.47	0.008
6-O-Me (EF)	Low strain	321.37	0.810
6-O-Me (EF)	High strain	5.18	0.073

Once the yield stress was overcome, the samples flowed very easily, as it can be observed from the low values in the power-law exponents in the high-strain regimes.

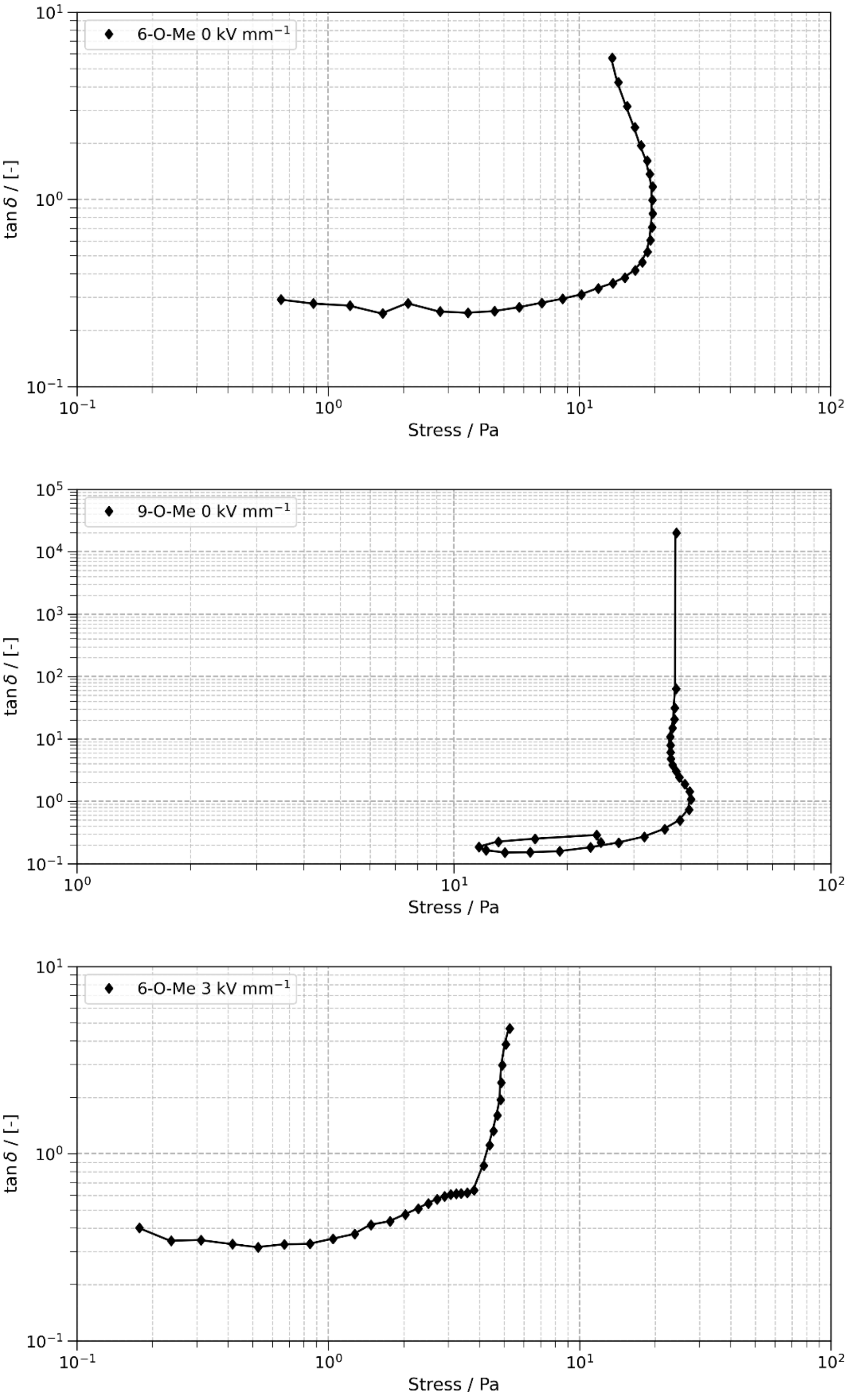

Figure S2. Loss factor ($tan\delta$) as a function of applied oscillatory stress obtained from amplitude sweep experiments performed at 1 rad s^{-1} and 25 °C for (a) 6-O-Me without an electric field, (b) 9-O-Me without an electric field, and (c) 6-O-Me under an applied electric field of 3 kV mm^{-1}. The sharp increase in tanδ was used as an additional indicator of the yielding transition.

Figure S3 presents the technical drawings corresponding to the microfluidic device 3D printed. For the printed geometry, the fast-printing setting of the Elegoo Mars 5 Ultra printer was used. The layer height and exposure time were set at 0.050 mm and 4.75 s, respectively. To ensure adhesion to the print plate, four bottom layers were applied with an exposure time of 60 s each.

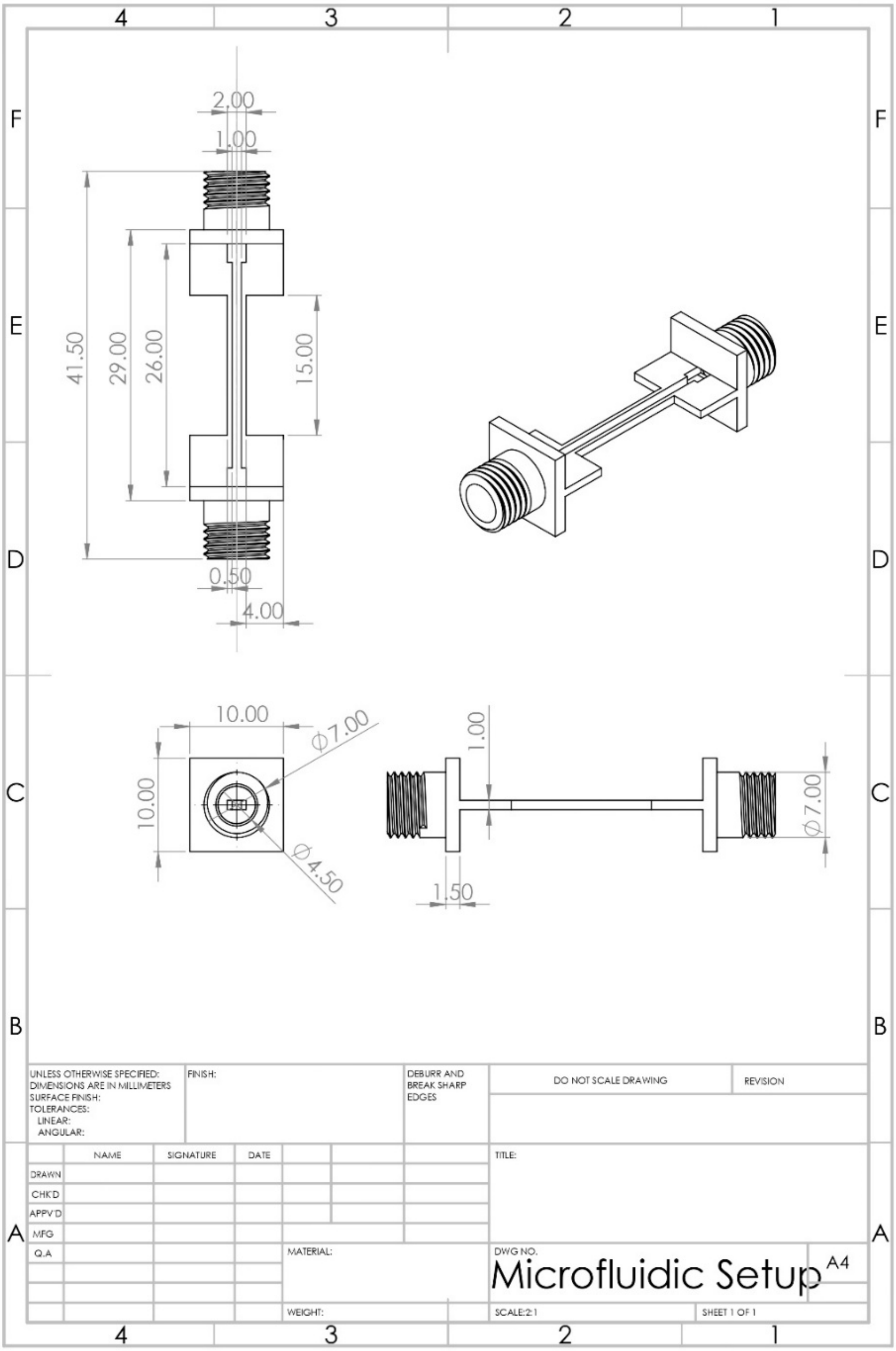

Figure S3. Technical schematic of the microfluidic fixture designed for loading gel samples with electric field application for microscopic analysis. Dimensions in mm.

Both geometries were printed lengthwise to prevent any residual support structures from interfering with the functionality of the parts, as illustrated for the microfluid setup in Figure S4.

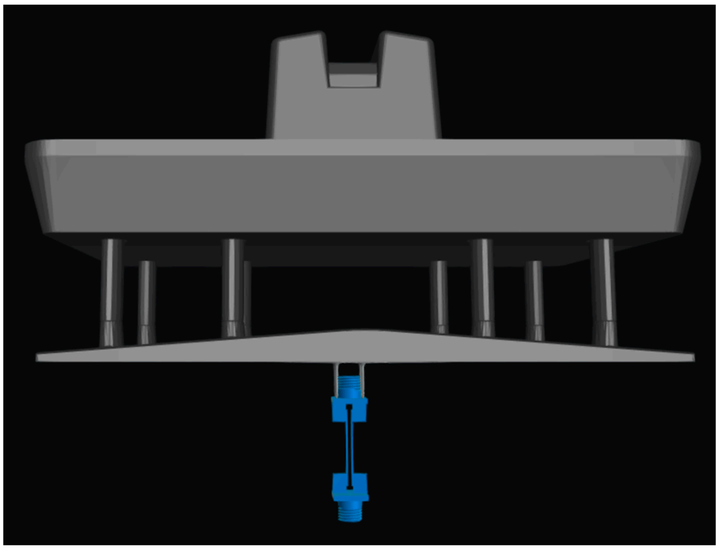

Figure S4. Front view of the microfluidic fixture in Chitubox Basic slicer, showing the printing direction (lengthwise).

The dimensional accuracy and print quality of the microfluidic fixture were first assessed by optical inspection. As shown in Figure S5, the top-view micrograph and corresponding technical drawing were used to compare the measured and designed dimensions of the printed features. Minor deviations from the intended geometry were observed. The flow channel’s widest section measured 1.830 mm versus the designed 2.000 mm, while the contracted region measured 0.844 mm compared to the intended 1.000 mm. The distance between the electrode slots, critical for accurate electric field application, was found to be 2.3 mm instead of 2.0 mm, yielding an effective field strength of approximately 1.3 kV mm^{-1} for a 3 kV potential difference. The device was printed lengthwise with a nominal layer height of 0.050 mm; however, measurements of five stacked layers yielded 0.266 mm, indicating slight inconsistencies in layer deposition along both the length and width axes.

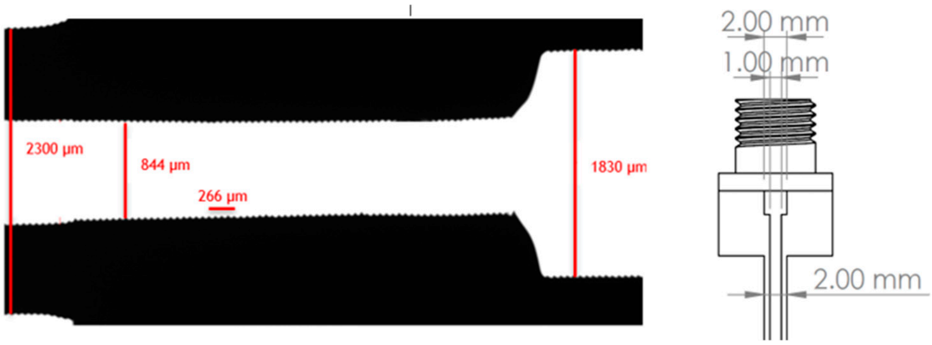

Figure S5. Microscopic top view image of the channel at 2.5 x microscope objective (left); technical drawing of the top view with key dimensions (right).

The inlet view of the geometry was also analyzed to verify the flow channel’s depth and cross-sectional uniformity (Figure S6). The measured channel depth was 0.569 mm, notably smaller

than the designed 1.000 mm, while the channel width from this perspective (0.987 mm) differed slightly from the top-view measurement (0.844 mm). This discrepancy likely originates from surface irregularities in the contraction zone, which produce non-uniform boundaries and locally increase the apparent channel width.

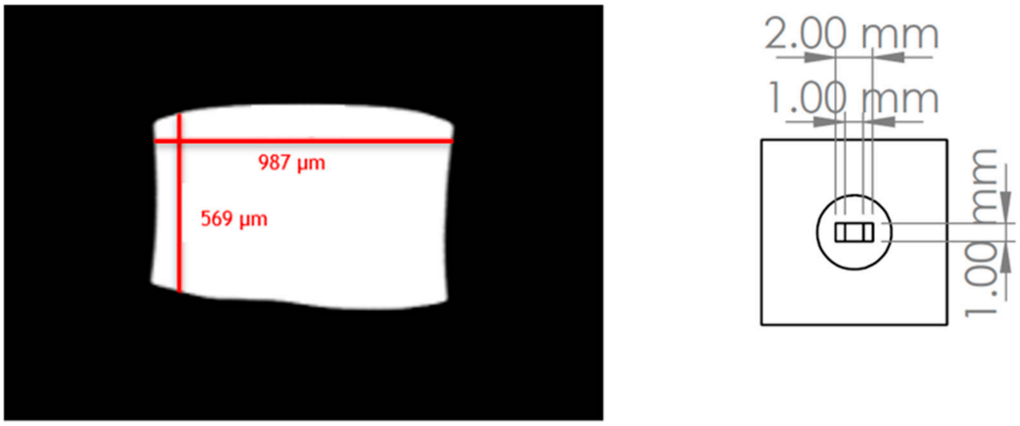

Figure S6. Microscopic inlet view image of the channel at 2.5 x microscope objective (left); technical drawing of the inlet view with key dimensions (right).

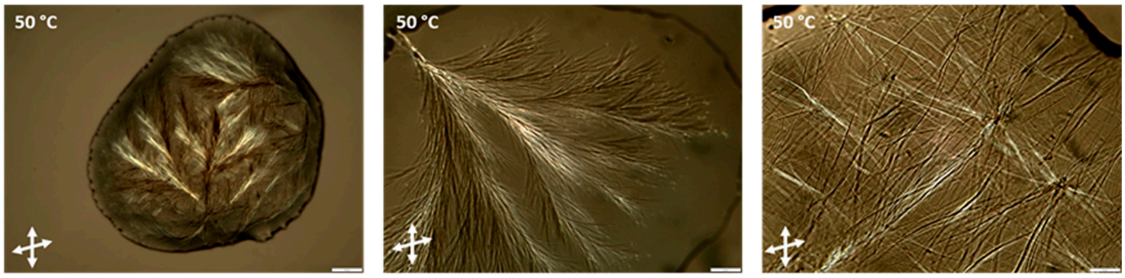

Figure S7. POM images of an uncovered sample of 6-O-Me/MBBA LC gels obtained on heating and subsequent cooling: (a) an electric field applied after gel formation at RT, (b) an electric field applied during cooling from the isotropic state through the gelation process to RT, and (c) without electric field, the white arrows present the position of the polarizers (scale bar 20 µm).

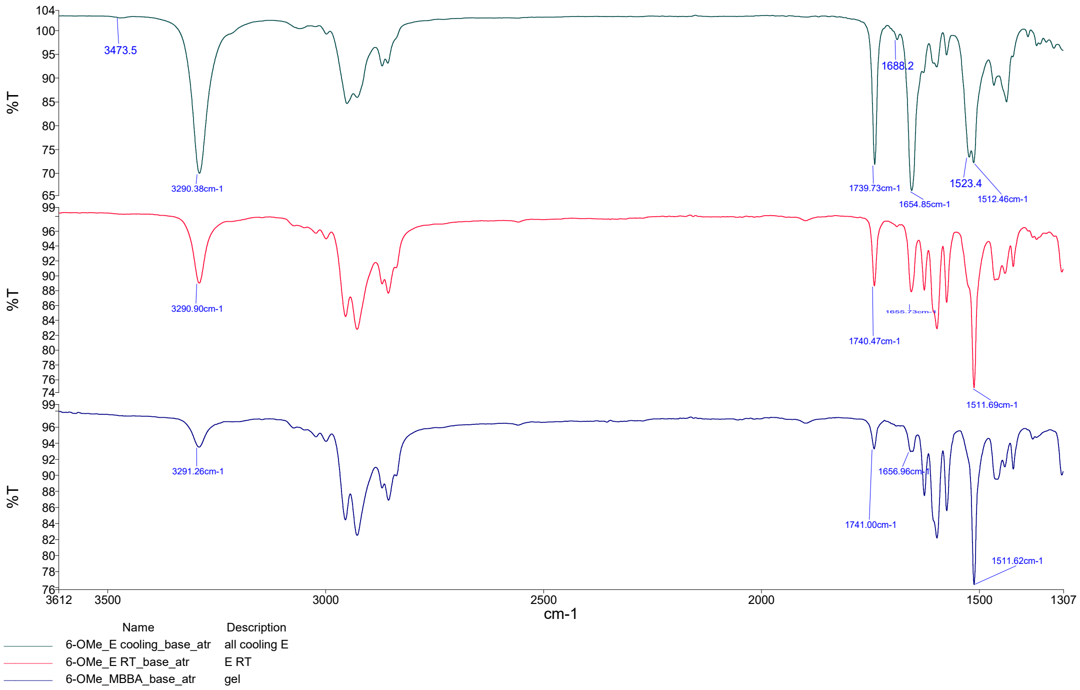

3473.5
3290.38cm-1
1739.73cm-1
1688.2
1654.85cm-1
1523.4
1512.46cm-1
3290.90cm-1
1740.47cm-1
1511.69cm-1
3291.26cm-1
1656.96cm-1
1741.00cm-1
1511.62cm-1
%T
cm-1
Name
Description
6-OMe_E cooling_base_atr
all cooling E
6-OMe_E RT_base_atr
E RT
6-OMe_MBBA_base_atr
gel

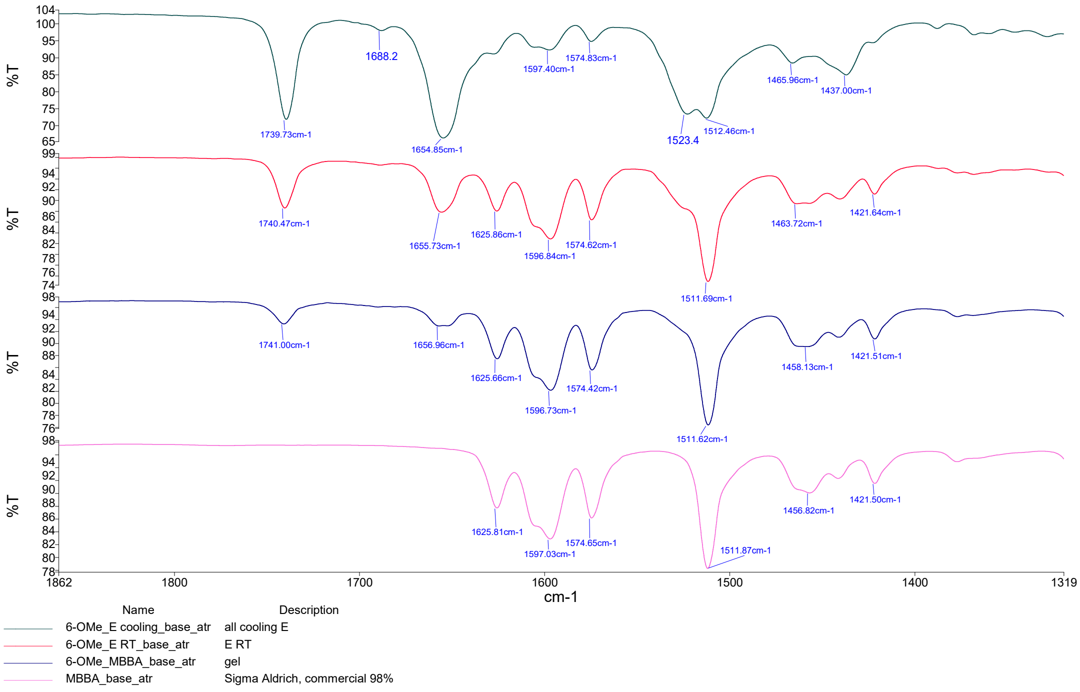

1739.73cm-1
1688.2
1654.85cm-1
1597.40cm-1
1574.83cm-1
1523.4
1512.46cm-1
1465.96cm-1
1437.00cm-1
1740.47cm-1
1655.73cm-1
1625.86cm-1
1596.84cm-1
1574.62cm-1
1511.69cm-1
1463.72cm-1
1421.64cm-1
1741.00cm-1
1656.96cm-1
1625.66cm-1
1596.73cm-1
1574.42cm-1
1511.62cm-1
1458.13cm-1
1421.51cm-1
1625.81cm-1
1597.03cm-1
1574.65cm-1
1511.87cm-1
1456.82cm-1
1421.50cm-1
%T
cm-1
Name
Description
6-OMe_E cooling_base_atr
all cooling E
6-OMe_E RT_base_atr
E RT
6-OMe_MBBA_base_atr
gel
MBBA_base_atr
Sigma Aldrich, commercial 98%

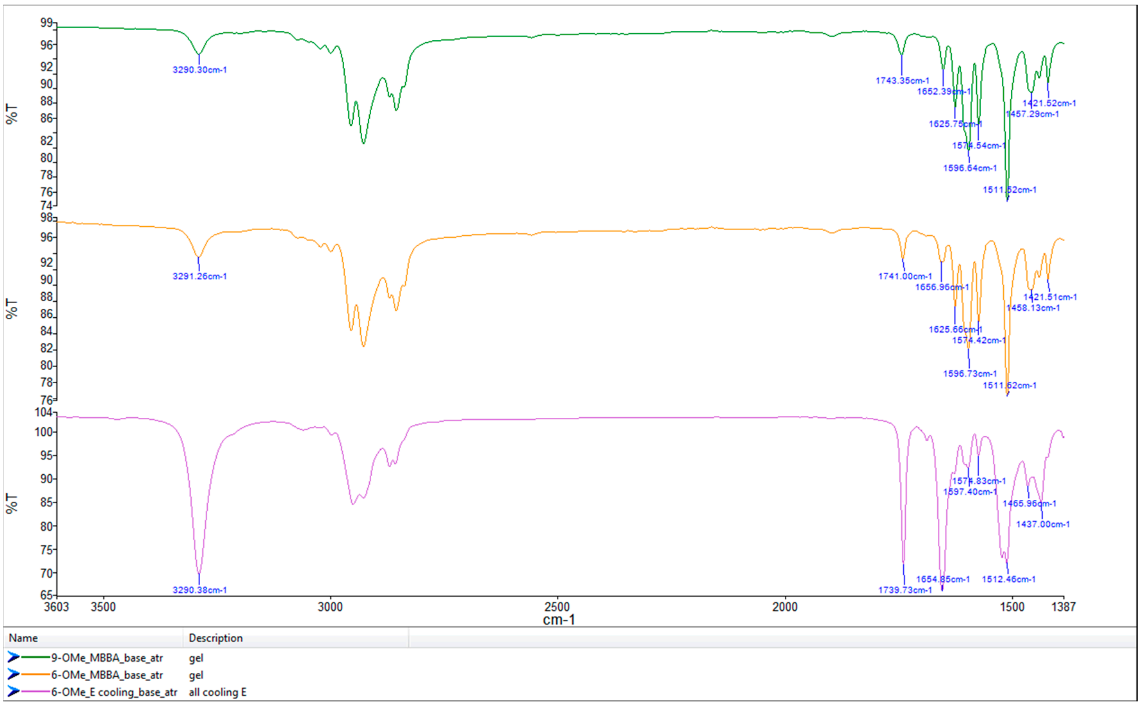

Figure S8. FTIR-ATR spectra of MBBA and 6-O-Me/MBBA and 9-O-Me/MBBA liquid-crystalline gels, including the 6-O-Me/MBBA sample exposed to an electric field after gel formation and the sample prepared under continuous electric-field-assisted cooling from the isotropic phase. Characteristic bands of hydrogen-bonded and free functionalities are indicated.
